# Late Metastatic Recurrence of Merkel Cell Carcinoma Nine Years After the Primary Diagnosis

**DOI:** 10.7759/cureus.94125

**Published:** 2025-10-08

**Authors:** Shelby L Kubicki, Jessica Forbes Kaprive, Andrew Kwong, Daniel Rivlin, Colleen M Emerson

**Affiliations:** 1 Dermatology, Indiana University School of Medicine, Houston, USA; 2 Dermatology, LewisGale Hospital Montgomery, Blacksburg, USA; 3 Mohs Micrographic Surgery, Larkin Community Hospital, South Miami, USA; 4 Mohs Micrographic Surgery, Larkin Community Hospital, Nova Southeastern University, South Miami, USA

**Keywords:** isolated cutaneous metastasis, merkel cell carcinoma (mcc), mohs micrographic surgery (mms), radiation therapy, skin cancer surveillance

## Abstract

Merkel cell carcinoma (MCC) is a rare, aggressive skin cancer of neuroendocrine origin that has been known to recur and metastasize within the first three years of initial diagnosis. MCC is typically diagnosed with clinicopathologic correlation of a red-purple nodule or flesh colored papule on sun-damaged skin and characteristic immunohistochemical staining. We present a case of a 66-year-old White man who was found to have metastatic MCC over nine years after the initial stage one tumor was diagnosed and treated with Mohs micrographic surgery (MMS) and radiation therapy. This case highlights the importance of defining prognostic biomarkers for each patient with MCC and consideration of extended surveillance beyond the current recommended three years. Clinicians should also be attentive when counseling patients to explain that the risk of late metastasis, although low, is still possible.

## Introduction

Merkel cell carcinoma (MCC) is a rare form of skin cancer of neuroendocrine origin that frequently presents as a red-purple or skin-colored papule or nodule on sun-damaged skin. This aggressive neoplasm most commonly presents on the head and neck region of elderly men and has high rates of recurrence and metastasis [1,2]. Key risk factors for the development of MCC include ultraviolet radiation, immunosuppression, advancing age, and Merkel cell polyomavirus (MCPyV) infection [3]. MCC is often diagnosed by tissue biopsy and immunohistochemistry with characteristic perinuclear staining with cytokeratin 20 (CK20) and other neuroendocrine markers. Staging modalities include imaging and sentinel lymph node biopsy. The mainstay of treatment is surgical excision of the primary tumor with possible adjuvant radiation [2]. The recurrence rate is approximately 40%, with 90% of recurrences arising within three years of primary tumor diagnosis [4,5]. Distant metastases occur in approximately one-third of patients [1]. Due to MCC's ability to induce a strong immune response, the tumor often develops resistance to therapy. As such, metastatic disease often portends a very poor prognosis [2]. For metastatic disease, systemic immune checkpoint inhibitors such as avelumab and pembrolizumab have become first-line agents since 2017 [1,6]. Avelumab, in particular, has been approved due to its improved survival outcomes in these patients who have failed one or more lines of chemotherapy [2]. Although chemotherapy was later found to be associated with worse overall survival, radiotherapy has been mostly well tolerated and associated with improved survival in patients with limited progression on avelumab [7,8].

## Case presentation

**Figure 1:**
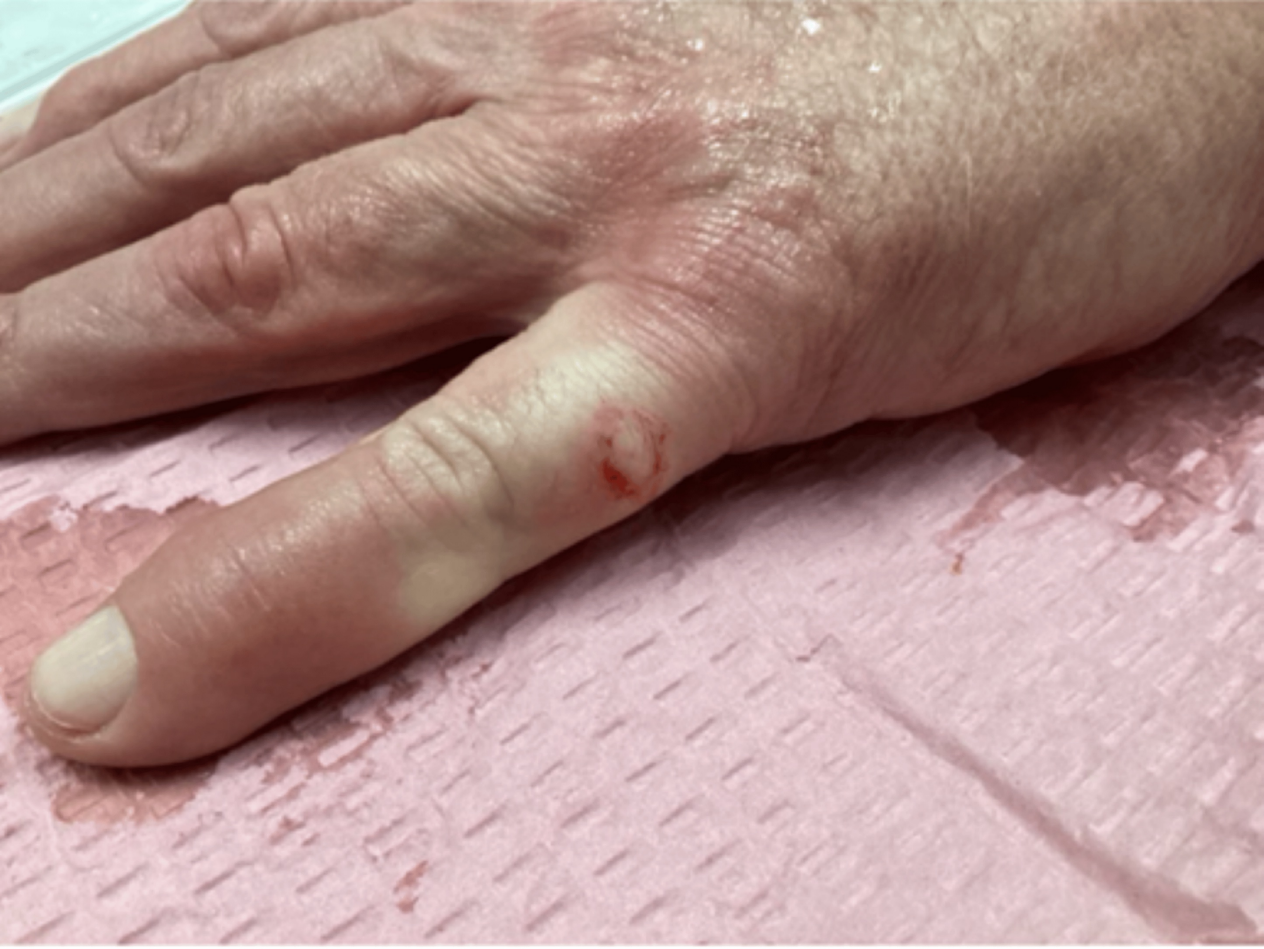
1.1 x 1.0 cm erythematous, firm nodule on the lateral aspect of the left, fifth proximal phalanx. Biopsy revealed small, monotonous blue cells with no connection to the epidermis indicating metastatic Merkel cell carcinoma.

**Figure 2:**
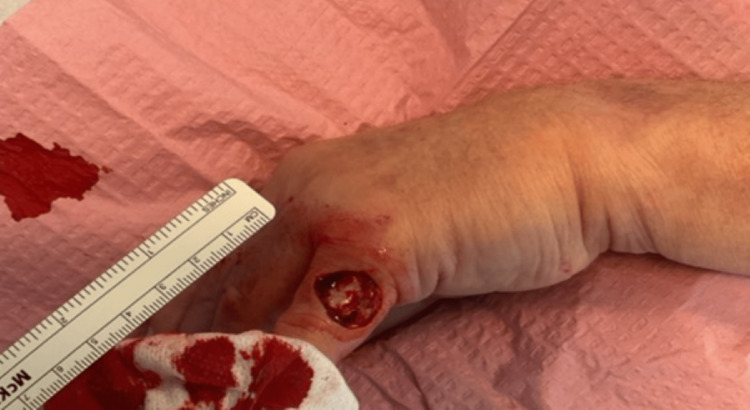
Immediately post-MMS of the metastatic MCC with negative margins after one stage, later confirmed with frozen section.

A 66-year-old White man developed a 2 x 1.5 cm pink firm nodule on the right ventral forearm. Biopsy and immunohistochemistry were consistent with MCC, showing sheets of monotonous, small blue cells and cytokeratin 20 (CK20) staining. The patient underwent Mohs micrographic surgery (MMS) with negative margins on frozen section analysis after one stage. Negative margins were confirmed with permanent sections. Following surgery, the patient received adjuvant radiation therapy to the primary site. The patient subsequently underwent full-body skin examination with palpation of the axillary lymph nodes every three months, with no evidence of recurrence or metastasis. Nine years and seven months after initial diagnosis, he developed a new 1.1 x 1 cm pink firm nodule on the left proximal phalanx of the lateral little finger (Figure 1). Biopsy revealed a dermal neoplasm of small blue cells with CK20 staining consistent with metastatic MCC, given history and location. MMS was performed with negative margins after one stage, on frozen sections confirmed by permanent section analysis (Figure 2). Positron emission tomography-computed tomography scan (PET-CT) showed no signs of lymph node involvement or additional metastasis, and the patient is currently pending radiation and systemic therapy.

## Discussion

We describe a case of metastatic MCC developing nine years after the primary tumor was diagnosed and treated. A recent study of 618 patients with recurrent MCC showed that 95% of recurrences arose within the first three years of primary diagnosis, with the latest recurrence occurring at 3.4 years, and an overall 5-year recurrence rate of 40% [4]. Similarly, MCC patients who develop distant metastases typically do so within the first several years of primary diagnosis. In a study of 215 patients with distant metastasis, 49% developed metastasis within one year of primary diagnosis and 80% within two years. To our knowledge, a recurrence or distant metastasis nine years after primary diagnosis has not been reported previously.

It is under investigation what the current high-risk factors for recurrence and metastasis are for MCC patients and the frequency of surveillance imaging. The current National Comprehensive Cancer Network (NCCN) guidelines recommend surveillance imaging and physical exams frequently for up to three years and then subsequent imaging if “clinically indicated” [9]. Patient baseline risk factors include the stage at diagnosis, with a higher risk of recurrence at a higher stage [4]. Our patient’s primary tumor was stage I at the time of diagnosis. Additionally, non-staging factors such as advanced age and other medical comorbidities should be taken into account in designing a patient’s surveillance period beyond the initial three to five years. This case highlights the importance of defining prognostic biomarkers for patients with MCC, with a surveillance period that may extend beyond three to five years. When counseling patients, it is also imperative to describe that the risk of late metastasis beyond five years, although low, is still possible. Other factors that affect surveillance timeframe include the status of polyomavirus titers. Polyomavirus-positive MCC tumors are associated with a longer recurrence-free survival compared with MCPyV-negative tumors [10]. Additionally, rising titers of MCPyV oncoprotein antibodies during the early surveillance period warrant more frequent intervals of imaging evaluation. It is currently unknown whether or not the MCPyV levels are helpful in predicting late recurrences or metastases. A multicenter study of 125 patients has highlighted the utilization of another known biomarker, including circulating tumor DNA (ctDNA), to help detect early recurrences in MCC patients and suggest it may be beneficial in monitoring long-term disease recurrence [11]. Therefore, this may be a screening modality to explore in the future.

## Conclusions

This case of metastatic MCC developing nine years after initial diagnosis underscores the potential for late recurrence and metastasis, which is extremely rare, given that the majority of recurrences occur within the first three years. Current guidelines recommend frequent surveillance for up to three years post-diagnosis. This case highlights the need for extended monitoring and patient counseling, especially considering non-staging factors such as advanced age and comorbidities. The role of prognostic biomarkers, including polyomavirus (MCPyV) status and circulating tumor DNA (ctDNA), is crucial in designing personalized surveillance strategies. This case emphasizes the importance of extended surveillance and tailored patient counseling to address the risk of late metastasis in MCC patients.

## References

[1] Lewis DJ, Sobanko JF, Etzkorn JR (2023). Merkel cell carcinoma. Dermatol Clin.

[2] Patel P, Hussain K (2021). Merkel cell carcinoma. Clin Exp Dermatol.

[3] Brady M, Spiker AM (2023). Merkel cell carcinoma of the skin. https://www.ncbi.nlm.nih.gov/books/NBK482329/.

[4] McEvoy AM, Lachance K, Hippe DS (2022). Recurrence and mortality risk of Merkel cell carcinoma by cancer stage and time from diagnosis. JAMA Dermatol.

[5] Singh N, McClure EM, Akaike T, Park SY, Huynh ET, Goff PH, Nghiem P (2023). The evolving treatment landscape of Merkel cell carcinoma. Curr Treat Options Oncol.

[6] Becker JC, Stang A, Schrama D, Ugurel S (2024). Merkel cell carcinoma: integrating epidemiology, immunology, and therapeutic updates. Am J Clin Dermatol.

[7] Vayntraub A, Tayeb N, Squires B (2021). The association of radiation therapy and chemotherapy on overall survival in Merkel cell carcinoma: a population-based analysis. Cureus.

[8] Ferini G, Zagardo V, Critelli P (2023). Introducing radiotherapy in metastatic Merkel cell carcinoma patients with limited progression on avelumab: an effective step against primary and secondary immune resistance?. J Pers Med.

[9] (2025). National Comprehensive Care Network Clinical Practice Guidelines in Oncology (NCCN guidelines): Merkel cell carcinoma, Version 2.2025. https://www.nccn.org/professionals/physician_gls/pdf/mcc_blocks.pdf.

[10] Harms KL, Zhao L, Johnson B (2021). Virus-positive Merkel cell carcinoma is an independent prognostic group with distinct predictive biomarkers. Clin Cancer Res.

[11] Akaike T, So N, Hippe DS (2022). The relationship between circulating tumor DNA with Merkel cell carcinoma tumor burden and detection of recurrence. J Clin Oncol.

